# Exploring the Bioactive Potential of *Pisolithus* (Basidiomycota): Comprehensive Insights into Antimicrobial, Anticancer, and Antioxidant Properties for Innovative Applications

**DOI:** 10.3390/microorganisms12030450

**Published:** 2024-02-23

**Authors:** Rui S. Oliveira, Marco Preto, Germana Santos, Ana Margarida Silva, Vitor Vasconcelos, Rosário Martins

**Affiliations:** 1 Centre for Functional Ecology, Associate Laboratory TERRA, Department of Life Sciences, University of Coimbra, Calçada Martim de Freitas, 3000-456 Coimbra, Portugal; rsoliveira@uc.pt; 2 Interdisciplinary Centre of Marine and Environmental Research (CIIMAR/CIMAR), University of Porto, Terminal de Cruzeiros do Porto de Leixões, Av. General Norton de Matos s/n, 4450-208 Matosinhos, Portugal; mpreto@ciimar.up.pt (M.P.); vmvascon@fc.up.pt (V.V.); 3 School of Health, Polytechnic Institute of Porto (ESS/P.PORTO), Rua Dr. António Bernardino de Almeida 400, 4200-072 Porto, Portugal; 10220896@ess.ipp.pt (G.S.); agl@ess.ipp.pt (A.M.S.); 4 Department of Biology, Faculty of Sciences, University of Porto (FCUP), Rua do Campo Alegre, Edifício FC4, 4169-007 Porto, Portugal

**Keywords:** biological activity, fungi, natural compounds, *Pisolithus arhizus*, *Pisolithus tinctorius*

## Abstract

Addressing pressing health concerns, modern medical research seeks to identify new antimicrobials to combat drug resistance, novel molecules for cancer treatment, and antioxidants for inflammation-related diseases. *Pisolithus* (Basidiomycota) is a ubiquitous and widely distributed fungal genus in forest ecosystems, known for establishing ectomycorrhizal associations with a range of host plants, enhancing their growth, and conferring protection against biotic and abiotic stresses. Beyond ecological applications, *Pisolithus* yields bioactive compounds with medicinal potential. This comprehensive review explores the transversal biological activity of *Pisolithus* fungi, aiming to provide a thorough overview of their antimicrobial, anticancer, and antioxidant potential. The focus is on elucidating bioactive compounds within *Pisolithus* to trigger further research for innovative applications. Compounds from *Pisolithus* displayed antimicrobial activity against a broad spectrum of microorganisms, including antibiotic-resistant bacteria. The efficacy of *Pisolithus*-derived compounds matched established medications, emphasizing their therapeutic potential. In anticancer research, the triterpene pisosterol stood out with documented cytotoxicity against various cancer cell lines, showcasing promise for novel anticancer therapies. *Pisolithus* was also recognized as a potential source of antioxidants, with basidiocarps exhibiting high antioxidant activity. In vivo validation and comprehensive studies on a broader range of compounds, together with mechanistic insights into the mode of action of *Pisolithus*-derived compounds, are compelling areas for future research.

## 1. Introduction

Nature stands as a highly promising reservoir of compounds with potential applications in human health. Some groups of organisms are widely known for their bioactive potential, namely plants, macroalgae, microalgae, bacteria, invertebrates, and fungi, particularly members of the Basidiomycota [1,2,3,4].

Basidiomycota is a diverse and ecologically significant phylum of fungi that encompasses over 40,000 species. They exhibit diverse lifestyles, from decomposers breaking down organic matter to pathogens affecting plant health. Additionally, some species function as mycorrhizal symbionts, forming vital partnerships with plants in various ecosystems [5,6]. Several species of this phylum reproduce sexually through basidiospores stored in the basidium, a club-shaped structure that gives these fungi the common name club fungi [7]. Basidiomycota, with their diverse properties, hold crucial applications in biotechnology and industry, contributing to areas such as enzyme production, bioremediation, and pharmaceutical development. Some well-known species of this phylum have been used for hundreds of years as food, such as *Lentinula edodes* (shiitake) and *Agaricus bisporus*, and as medicines (e.g., *Ganoderma lucidum* and *Wolfiporia extensa*) [8,9,10]. Others are known for their poisonous nature, like the death cap (*Amanita phalloides*) [11,12]. The number of bioactive natural products from Basidiomycota is considerable, as recently reviewed [8,13,14,15,16,17], and includes compounds with antimicrobial, anticancer, antioxidant, anti-inflammatory, and nutraceutical properties, among others. 

*Pisolithus*, a fascinating genus of fungi within the Basidiomycota, exemplifies the diverse ecological roles and bioactive potential found within this group of fungi. *Pisolithus* species are widely distributed across various ecosystems on six continents and are recognized for their distinctive fruiting bodies (basidiocarps). Characterized by large, round structures resembling puffballs, *Pisolithus* basidiocarps are often found partially buried in the soil. Besides the basidiocarps, *Pisolithus* are composed of hyphae, thread-like structures that make up the mycelium of the fungus, and spores that are produced in the basidiocarps [18,19,20]. One of the notable features of *Pisolithus* lies in the fact that its species form ectomycorrhizal associations with plants, particularly trees. The *Pisolithus* genus contains 19 species that colonize the roots of more than 50 host plants. The mycorrhizal partnership involves a mutually beneficial relationship where the fungus assists the plant in nutrient uptake, especially phosphorus, in exchange for carbohydrates produced by the plant through photosynthesis. This symbiotic association enhances the growth and protects the plant against biotic and abiotic stresses [19,21,22]. The beneficial effects of *Pisolithus* species lead to their incorporation into forestry management plans for promoting healthier ecosystems and enhancing the long-term sustainable productivity of forestry plantations [23,24,25,26]. The most studied *Pisolithus* species is *Pisolithus arhizus* (syn. = *Pisolithus tinctorius*). 

Beyond their ecological role and forestry applications, researchers have explored bioactive compounds produced by *Pisolithus* species, discovering substances with antimicrobial, anticancer, and antioxidant properties. These findings open avenues for further exploration into the medicinal potential of *Pisolithus*-derived compounds. 

In contemporary medical research, critical challenges abound, propelling the field toward innovative solutions. The exploration and identification of novel antimicrobial products are imperative in combatting rising drug resistance [27,28]. Moreover, the ongoing search for new natural molecules in cancer treatment reflects a pressing need for transformative approaches to enhance therapeutic outcomes in the relentless battle against cancer [29,30]. Simultaneously, the quest for antioxidant molecules effective against inflammation-related diseases addresses widespread health concerns [31,32]. 

**Figure 1:**
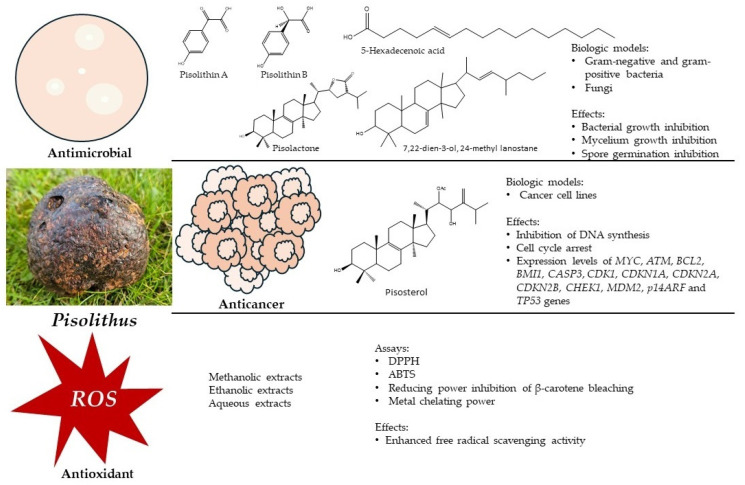
A schematic representation of the bioactivities of *Pisolithus*, as described in this review.

In this context, and considering the transversal biological activity of *Pisolithus*, the objective of this review is to provide a comprehensive overview of the (i) antimicrobial, (ii) anticancer, and (iii) antioxidant potential of these fungi. The focus extends to highlighting the bioactive compounds isolated from *Pisolithus* with the aim of catalyzing further research for exploring innovative applications of these ubiquitous and widely distributed fungi (Figure 1).

## 2. The Bioactive Potential of *Pisolithus*

### 2.1. Antimicrobial Activity

Each year, millions of people worldwide succumb to microbial infections [33,34]. Microbial resistance to antimicrobial drugs is a growing global public health concern. Overuse and misuse of antibiotics have led to the development of resistant strains, rendering once-effective treatments ineffective [27,35]. Pathogenic microorganisms are a challenging threat to both human and animal health, underscoring the critical importance of addressing microbial infections in the context of public health [36,37]. Phytopathogens are harmful organisms that cause plant diseases, posing a significant threat to agriculture. They can devastate crops, leading to yield losses, economic challenges, and food security concerns [38,39]. Consequently, also in this field, an immediate and imperative need arises for the exploration and identification of novel antimicrobial agents. Recently, two new phenols with activity against several plant pathogenic fungi and clinically important bacterial strains were obtained from fungi [40].

**Figure 2:**
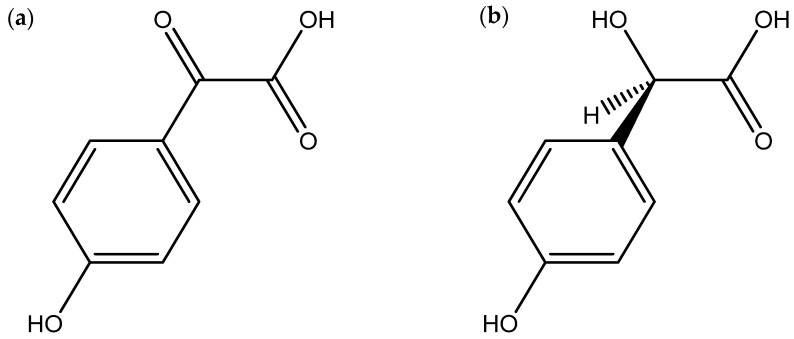
Chemical structures of pisolithin A (**a**) and pisolithin B (**b**), two *Pisolithus*-derived phenolic compounds with antimicrobial activity.

Antimicrobial compounds exert their effects through various mechanisms, such as inhibition of the synthesis of the cell wall, proteins, nucleic acids, and cell membrane disruption of target microorganisms [41]. Several studies documented in the literature highlight *Pisolithus* as a potential genus with antimicrobial activity (Table 1). Kope and Fortin [42] demonstrated that the mycelium of *P. tinctorius* has antifungal activity by inhibiting spore germination, provoking hyphal lysis, and inhibiting chitin synthesis and consequently disrupting the cell wall in a range of phytopathogenic fungi, including *Rhizoctonia praticola*, *Truncatella hartigii*, *Sphaerosporella brunnea*, *Fusarium solani*, *Brunchorstia pinea*, and *Cochliobolus sativus*. Subsequently, the compounds pisolithin A and pisolithin B (Figure 2) were isolated from *P. arhizus* mycelium [43]. These two phenolic compounds showed a considerable capacity for reducing the mycelial growth of several phytopathogenic fungi, such as *Rhizoctonia solani*, *Verticillium dahlia*, *Pyrenochaeta terrestris*, *Cochliobolus sativus* and *Septoria musiva*, phytopathogenic oomycetes, such as *Pythium debaryanum* and *Pythium ultimum* and dermopathogenic fungi, such as *Microsporum gypseum* and *Trichophyton equinum*. In some cases, the inhibition was higher than that obtained with other antifungal agents such as nystatin and polyoxin D. With the compound pisolithin A, it was also demonstrated a spore germination inhibition of the plant pathogen *T. hartigii*, in concentrations ranging from 50 to 150 µg/mL in 24 h [44]. Shrestha et al. [45] showed that *Pisolithus* sp. extracts were able to inhibit the growth of the bacterial pathogens *Klebsiella* sp. and *Escherichia coli*. Another example that reinforces the antimicrobial proprieties of *Pisolithus* is the work of Ameri et al. [46], who conducted bioassays with *Pisolithus albus* crude extracts and isolated fractions against strains of methicillin-resistant *Staphylococcus aureus*. In their study, the authors observed a strong antibiotic action, especially in the fraction that contained sesquiterpenes. *Pisolithus microcarpus* crude extracts have also shown antibacterial potential against pathogens such as *Pseudomonas aeruginosa* and *S. aureus* [47].

In another study, ethanolic extract of *P. albus* displayed antibacterial activity against Gram-positive (*Bacillus subtilis*, *Enterococcus faecalis*, *Listeria monocytogenes*, *S. aureus*) and Gram-negative bacteria (*Aeromonas hydrophila*, *E. coli*, *P. aeruginosa*, *S. typhimurium*) [48]. 

In a recent investigation involving multidrug-resistant Gram-positive and Gram-negative bacteria isolated from wound exudates of hospitalized human patients, Martins et al. [49] found that hydroethanolic extracts of *P. tinctorius* had minimum inhibitory concentrations (MICs) of 5, <0.156, 10, 5, 5, 5 mg/mL for *Enterococcus faecium*, *S. aureus*, *Acinetobacter baumannii*, *Enterobacter aerogenes*, *Klebsiella pneumoniae*, and *P. aeruginosa*, respectively.

**Figure 3:**
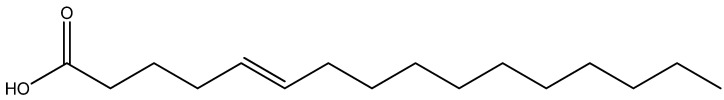
Chemical structure of 5-hexadecenoic acid, a secondary metabolite with antibacterial activity isolated from *Pisolithus tinctorius*.

Carmo [50] prepared hexane and ethyl acetate fractions from *P. tinctorius* basidiocarps. Both fractions inhibited the growth of the Gram-positive bacterium *Enterococcus* sp., with MICs of 125 and 62.5 μg/mL for hexane and ethyl acetate fractions, respectively. The hexane fraction also inhibited the growth of *Bacillus cereus* and *S. aureus*, both with a MIC of 125 μg/mL. The human pathogenic Gram-negative bacteria, *Shigella sonnei* and *Shigella flexneri*, were also inhibited by these fractions. The antifungal potential of both fractions was demonstrated against human pathogenic fungi, with MICs of 125 μg/mL for *Cryptococcus neoformans* and *Cryptococcus gattii* and 62.5 μg/mL for *Candida krusei*. Following these promising results, Carmo [50] isolated and identified four secondary metabolites from the *P. tinctorius* extracts and tested the antimicrobial activity of these compounds separately (Table 1). The compounds were a ceramide P56, 5-hexadecenoic acid, and two triterpenoids: pisolactone and the new metabolite 7,22-dien-3-ol, 24-methyl lanostane. Although all four compounds displayed a certain degree of antibacterial activity, 5-hexadecenoic acid (Figure 3) was notable for inhibiting the pathogenic Gram-negative bacteria *S. sonnei* and *E. aerogenes* (MIC 25 μg/mL), *P. aeruginosa*, *Morganella morgani*, *K. pneumoniae* and *S. flexneri* (MIC 50 μg/mL), and the Gram-positive bacteria *B. subtilis* (MIC 50 μg/mL) and *S. aureus* (MIC 12.5 μg/mL).

The antimycobacterial capacity was also revealed by pisolactone and ceramide P56, which were effective against the pathogenic *Mycobacterium abcessus*, exhibiting MIC values of 31.25 and 15.62 μg/mL, respectively. Moreover, the MIC displayed by ceramide P56 against *M. abcessus* was equivalent to the widely used antibiotic clarithromycin (MIC 16 μg/mL). Ceramide P56 was also effective against *Mycobacterium fortuitum*, which is commonly associated with infections affecting the skin, soft tissues, and bones. The obtained MIC of 31.25 μg/mL was equivalent to that of sulfametoxazol (MIC 32 μg/mL), a commonly prescribed antibiotic [50]. 

**Figure 4:**
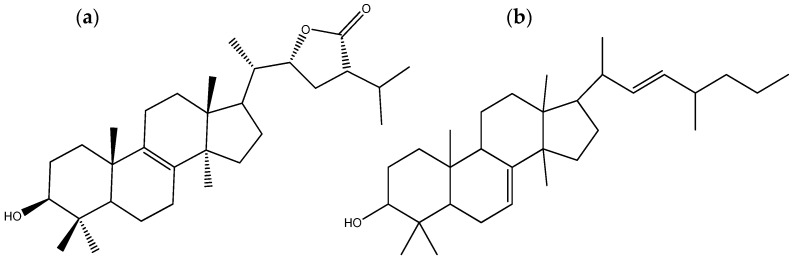
Chemical structures of the triterpenoids pisolactone (**a**) and 7,22-dien-3-ol, 24-methyl lanostane (**b**), two secondary metabolites with antifungal and antibacterial activity isolated from *Pisolithus tinctorius*.

All four isolated compounds of *P. tinctorius* presented antifungal activity, with the most significant being pisolactone (Figure 4a) and 7,22-dien-3-ol, 24-methyl lanostane (Figure 4b). The MICs displayed by these two compounds against *Candida tropicalis*, *C. krusei*, *C. neoformans*, *C. gattii*, and *Candida glabrata* were 6.25, 1.56, 50, 50, and 1.56 μg/mL, respectively. The MICs of pisolactone and 7,22-dien-3-ol, 24-methyl lanostane against the three *Candida* species were lower than that of fluconazole, an antifungal medication commonly used to treat fungal infections such as vaginal yeast infections (candidiasis), oral and esophageal thrush, cryptococcal meningitis, and other systemic fungal infections. Additionally, the MICs of pisolactone and 7,22-dien-3-ol, 24-methyl lanostane against *C. krusei* and *C. glabrata* were equal to those of nystatin, another widely used antifungal medication to treat fungal infections, particularly those caused by the yeast *Candida* [50]. These findings indicate the potential of the two *Pisolithus*-derived compounds to be used as novel antifungal drugs.

**Table 1:** *Pisolithus* extracts, fractions, and isolated compounds and their antimicrobial activities.

Extract/Compound	Species	Fungal Structure	Assay	Antimicrobial Activity	Reference
Pisolithin A Pisolithin B (phenolic compounds)	*P. arhizus*	Mycelium	Spore germination Hyphal growth measured by protein estimation	Activity against phytopathogenic fungi, phytopathogenic oomycetes, and dermopathogenic fungi	[43,44]
Pisolactone (triterpenoid)	*P. tinctorius*	Basidiocarp	Broth microdilution	Activity against Gram-negative and Gram-positive bacteria Antimycobacterial Antifungal	[50]
7,22-dien-3-ol, 24-methyl lanostane (triterpenoid)	*P. tinctorius*	Basidiocarp	Broth microdilution	Activity against Gram-negative and Gram-positive bacteria Antifungal	[50]
5-hexadecenoic acid (unsaturated fatty acid)	*P. tinctorius*	Basidiocarp	Broth microdilution	Activity against Gram-negative and Gram-positive bacteria Antifungal	[50]
Ceramide P56 (ceramide)	*P. tinctorius*	Basidiocarp	Broth microdilution	Activity against Gram-negative and Gram-positive bacteria Antimycobacterial Antifungal	[50]
Crude extracts/fractions	*P. albus*	Basidiocarp	Agar well diffusion	Activity against methicillin-resistant *Staphylococcus aureus*	[46]
Methanolic extracts/fractions	*P. microcarpus*	Mycelium	Disk diffusion	Activity against *Pseudomonas aeruginosa* and *S. aureus*	[47]
Ethanolic extracts	*P. albus*	Basidiocarp	Disk diffusion	Activity against Gram-negative and Gram-positive bacteria	[48]
Ethyl acetate extracts	*P. tinctorius*	Basidiocarp and spores	Agar well diffusion	Activity against phytopathogenic fungi	[51]
Ethyl acetate extracts	*P. tinctorius*	Mycelium filtrate	Mycelium growth in solid medium	Activity against phytopathogenic fungi	[52]
Hydroethanolic extracts	*P. tinctorius*	Basidiocarp	Broth microdilution	Activity against multidrug-resistant Gram-negative and Gram-positive bacteria	[49]

### 2.2. Anticancer Activity

According to the World Health Organization global cancer statistics (GLOBOCAN), in 2020, around 19 million new cancer cases were diagnosed, and over 9.9 million deaths were registered [53]. Although research into cancer treatment has been insistent, the number of diagnosed cases is expected to keep increasing, adding up to 29.4 million cases by 2040 [54]. The conventional approach to cancer treatment involves chemotherapy, which is highly associated with aggressive and prolonged collateral effects, mainly due to the very toxic nature of the involved compounds. Accordingly, the search for alternative and less toxic approaches has gained prominence, encouraging increased research into the discovery and development of new products, namely from natural origin [55,56]. 

Anticancer compounds exert their effects through various mechanisms to target cancer cells and inhibit tumor growth. Some common mechanisms of action include inhibition of DNA synthesis, cell division and angiogenesis, induction of apoptosis, and disruption of tumor metabolism [57].

**Figure 5:**
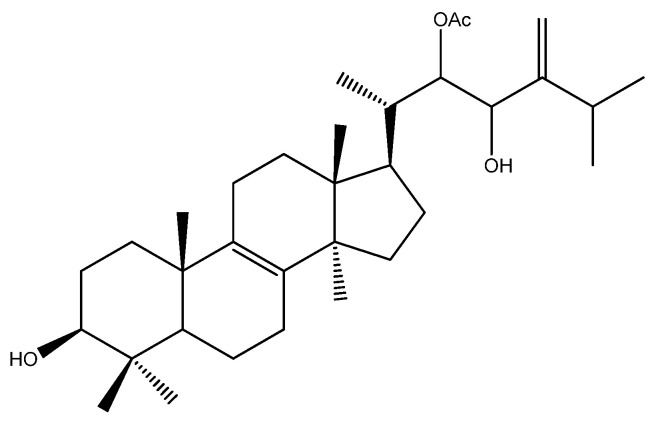
Chemical structure of pisosterol, a *Pisolithus*-derived triterpene with anticancer activity.

Also, in the field of natural compounds for cancer treatment, the genus *Pisolithus* has aroused interest, with a focus on the triterpene pisosterol. Pisosterol was first isolated from the basidiocarps of *P. tinctorius* and chemically elucidated by Gill et al. [58] (Figure 5). Over the years, its cytotoxicity and mechanisms of action have been elucidated, mainly in cellular models, with relevant results (Table 2).

One of the first reports on the anticarcinogenic action of pisosterol was ascribed to Montenegro and co-workers in 2004 [59]. The effect of the compound was tested in vitro, in mouse erythrocytes to infer its effects on membrane disruption, sea urchin developing embryos, and in the tumor cell lines CEM (human leukemia), HL-60 (human leukemia), B16 (murine melanoma), HCT-8 (human colon cancer), MCF-7 (human breast cancer), PC-3 (human prostate cancer) and SF-268 (human neuroblastoma) for cytotoxicity. The cytotoxicity of the compounds inferred using the viability assay based on the reduction of the 3-(4,5-dimethylthiazol-2-yl)-2,5-diphenyltetrazolium bromide salt (MTT) by viable cells was compared to doxorubicin and etoposide, which are clinically used drugs in cancer chemotherapy. The results showed no activity in erythrocytes or even in the development of the embryos of sea urchins. However, there was a strong growth inhibition in all tumor cell lines, notably in leukemia CEM, HL-60 cells, and B16 melanoma cells with IC_50_ of 1.55, 1.84, and 1.65 μg/mL, respectively. Sea urchins have been used as model species to infer mechanisms of cell cycle control, cell adhesion, fertilization, cell differentiation, gene expression regulation, and death [60], and in general, cytotoxic substances tested in the sea urchin eggs and tumor cells are active in both assays [61]. This last assumption was not observed by Montenegro et al. [59] in their work with pisosterol. Since phase G1 is arrested in sea urchin cells and G2 is briefer than in mammals, the authors suggested an action of pisosterol on these cell cycle phases.

Given the cytotoxic results induced by pisosterol and described in the previous research, Montenegro et al. [62] conducted a study to determine if pisosterol was able to induce cell differentiation using the leukemia cell line HL-60 as a cancer cell model and peripheral blood mononuclear cells (PBMCs) as non-cancer cells. The data showed that HL-60 cells treated with pisosterol tend to differentiate into monocytic cells, and apoptosis was detected. No cytotoxicity was registered in PBMCs, even in the highest concentration (5 μg/mL), suggesting that pisosterol can be selective to cancer cell lines. In addition, Burbano et al. [63] conducted a study to elucidate the mechanisms of action of pisosterol in HL-60 leukemia cells by analyzing the homogeneously staining region (HSR) 8q24 aberration. Chromosome 8 rearrangements showing HSRs are recurrent karyotype abnormalities predominantly shown by HL-60 cells. The authors found that 99% of cells showed HSRs before pisosterol treatment, while 90% of the analyzed cells lacked this HSR region when treated with pisosterol. Although HL-60 cells resumed their growth after washing and re-incubation in a pisosterol-free culture medium and cells with HSRs did not suffer significant apoptosis or necrosis in the presence of pisosterol at a concentration of 1.8 μg/mL, the results revealed pisosterol as a putative drug to be used in combination with conventional anticancer therapy.

Pereira and co-authors [64] described the effect of pisosterol in the glioblastoma multiform (GBM) cell lines U343 and AHOL1. The treatment with three concentrations of pisosterol (0.5, 1.0, and 1.8 μg/mL) did not alter the cell morphology of the two cell lines, which is an indication that pisosterol does not induce cell differentiation in these GBM cells. Results showed a significant decrease in mitotic index only at 1.8 mg/mL. These results corroborate the findings of Montenegro et al. [59], in which the pisosterol IC_50_ for HL-60 cell was 1.84 μg/mL as previously described. Cells were also treated for abnormalities involving chromosome 8 or 8q24, the location where the *C-MYC* gene is mapped. The results indicated that no new chromosomal abnormalities emerged after treatment, suggesting that pisosterol has no clastogenic and/or aneugenic effect. This finding corroborates the earlier work of Burbano et al. [63], which was also conducted on HL-60 cells. Still, in the work of Pereira et al. [64], the interphase nuclei of the U343 and AHOL1 cells were analyzed by fluorescence in situ hybridization (FISH) for *C-MYC* before and after treatment with pisosterol. Results showed that after treatment with 1.8 μg/mL pisosterol, only 33% of U343 cells and 15% of AHOL1 cells had more than two *C-MYC* alleles when compared to the 72% of U343 and 65% of AHOL1 cells before the treatment. This result might be of particular importance since the *C-MYC* protein is known to be involved in cell cycle progression from G1 to S phase.

The potential of pisosterol in cancer therapy was also described on a panel of glioma cell lines by Ferreira et al. [65]. Cellular viability and proliferation of U343, AHOL1, U-87MG, and 1321N1 cells were significantly decreased in a dose-dependent manner (concentrations of 0.97, 1.94, and 3.50 μM), with inhibition of cell proliferation via the G2/M phase arrest and cell death by apoptosis. Treatment with pisosterol also revealed a dose-dependent downregulation of the expression of *MYC*, *BCL2*, *BMI1*, and *MDM2* genes and a significant dose-dependent upregulation of gene expression levels of *CASP3*, *TP53*, *ATM*, *CDK1*, *CDKN1A*, *CDKN2A*, *CDKN2B*, *CHK1* and *p14ARF*, which corroborate the inhibition of cell cycle progression and both the caspase-independent and caspase-dependent apoptotic pathways (Table 2). 

While the previously described studies on the anticancer potential of pisosterol were based on cancer cell assays, in 2008 Montenegro and colleagues [66] conducted a study directed to an in vivo evaluation. In the study, sarcoma 180 tumor cells were subcutaneously transplanted into Swiss female mice and treated with 50 and 100 mg/m^2^ of pisosterol for 7 days. The results showed a tumor growth inhibition ratio of 43.0 and 38.7% for mice treated with pisosterol at 50 and 100 mg/m^2^, respectively, and 54.9% for mice treated with 5-fluorouracil at 50 mg/m^2^ as positive control. In order to evaluate the toxicological impact of pisosterol in in vivo models, morphological analyses were made. The treatment had an impact on the liver, showing Kupffer cells hyperplasia, focal infiltrate of inflammatory cells, and centrilobular venous congestion, demonstrating that the liver is a target organ of pisosterol. On the other hand, the authors concluded that the damages can be reversible since no stromal fibrosis was detected and conjunctive tissue was preserved.

Although the most relevant anticancer studies on *Pisolithus* were assigned to pisosterol, several other compounds have been described. Different medium and long-chain saturated fatty acids that have been isolated from methanolic extracts of *P. tinctorius* [67] were indicated to have anticancer activity, the most relevant being capric and lauric acids. These compounds were shown to induce apoptosis in colorectal, skin, and breast cancer cell lines [68,69,70]. Lastly, the ergosterol derivate, ergosterol peroxide that had been isolated from basidiocarps of *P. tinctorius* among other fungi [71], showed promising results against different cancer cell lines such as HT29 colon adenocarcinoma cells [72]. More recently, Parisi and co-authors [73] reported the isolation and structure elucidation of thirteen new and two already known triterpenoids from chloroform and methanolic extracts of basiodiocarps of *P. arhizus*. From the panel of isolated compounds, 24-methyllanosta-8,24 (31)-diene-3β,22ε-diol previously described by Baumert et al. [74] and the newly isolated 24 (31)-epoxylanost-8-ene-3β, 22S-diol were found to induce moderate cytotoxicity in a dose-dependent manner on the cancer cell lines U-87MG and Jurkat, with no cytotoxicity in the normal keratinocytes cell line HaCaT. 

Most of the anticancer studies directed to *Pisolithus* have been based on compounds isolated from the basiodiocarp. In a pioneering work, Alves et al. [75] evaluated the anticancer potential of DCM/MeOH and EtOAc/MeOH crude extracts of *P. tinctorius* spores against the human osteosarcoma cell line MG63, the human breast carcinoma cell line T47D, the human colon adenocarcinoma cell line RKO, and the normal human brain capillary endothelial cell line hCMEC/D3. The cytotoxicity results based on the MTT assays showed a decrease in cancer cell line viability of 92% and 88% for DCM/MeOH and EtOAc/MeOH crude extracts, respectively, without a significant reduction in the viability of the normal cells. The most notable results were obtained with the DCM/MeOH extract with cell viability of 12% in RKO and MG63 cells and 6% in T47D cells after 48 h of exposure. These results extend the anticancer activity of *Pisolithus* to structures other than the basiodiocarp, highlighting the potential of this fungal genus.

**Table 2:** Anticancer activity of the triterpene pisosterol isolated from *Pisolithus tinctorius*.

Biological Model	Assay	Mechanism/Effect	Reference
CEM, HL-60, B16, HCT-8, MCF-7, PC-3, SF-268 cell lines	MTT	Cytotoxicity	[59]
HL-60 cell line	MTT	Cytotoxicity	[62]
	Trypan blue exclusion	Viability	
	α-Naphthyl acetate esterase activity	Cell differentiation	
	NBT		
	BrdU incorporation	Inhibition of DNA synthesis	
	Differential fluorescent staining with acridine/orange ethidium bromide	Apoptosis	
	Mitotic index	Cell cycle arrest	[63]
	Chromosome analysis	Homogeneously staining region (HSR) 8q24 aberration	
U343, AHOL1 cell lines	Cytogenetic characterization: metaphases stained with Giemsa solution and banded with trypsin-Giemsa Mitotic index FISH analysis	8q24.12–q24.13 chromosome aberrations Cytotoxicity Expression of *C-MYC* gene	[76]
U343, AHOL1, U-87 MG, 1321N1 cell lines	MTT	Cytotoxicity	[65]
	Trypan blue exclusion	Viability	
	Flow cytometry	Cell cycle arrest	
	Staining with Annexin V-FITC/PI	Apoptosis	
	qPCR and western blotting	Expression levels of *MYC*, *ATM*, *BCL2*, *BMI1*, *CASP3*, *CDK1*, *CDKN1A*, *CDKN2A*, *CDKN2B*, *CHEK1*, *MDM2*, *p14ARF* and *TP53* genes	
Swiss female mice	Histopathology and morphologic observations	Induction of cellular and nuclear pleomorphism; coagulative type necrosis	[66]
MG63, T47D, RKO cell lines	MTT	Cytotoxicity	[75]

MTT—3-(4,5-dimethylthiazol-2-yl)-2,5-diphenyltetrazolium bromide salt; NBT—nitro blue tetrazolium salt; CEM—T lymphoblast cell line; B16—murine melanoma cell line; HCT-8—human colon adenocarcinoma cell line; MCF-7—breast cancer cell line; PC-3—prostate cancer cell line; SF-268—human neuroblastoma cell line; HL-60—human leukemia cell line; U343—human glioblastoma cell line; AHOL1—human glioblastoma cell line; U-87 MG—human glioblastoma cell line; 1321N1—human astrocytoma cell line; MG63—human osteosarcoma cell line; T47D—human breast carcinoma cell line; RKO—human colon adenocarcinoma cell line; FISH—fluorescence in situ hybridization; FITC/PI—fluorescein isothiocyanate/propidium iodide; qPCR—quantitative polymerase chain reaction; DNA—deoxyribonucleic acid.

### 2.3. Antioxidant Activity

More than 50% of global deaths are linked to inflammation-related diseases, such as stroke, diabetes, cancer, and neurodegenerative and autoimmune conditions [31,77]. 

Inflammation is a natural defense mechanism against endogenous or exogenous antigens and involves enhanced or exacerbated production of reactive nitrogen species (RNS) and reactive oxygen species (ROS). However, ROS and RNS are generated in regular metabolic pathways by most cells, as well as in inflammatory processes to eliminate pathogens when, in excess, these oxidative species can promote oxidative stress associated with chronic inflammation [78]. Antioxidants play a pivotal role in maintaining cellular homeostasis and preventing oxidative stress-induced damage. They are compounds that neutralize or inhibit the detrimental effects of ROS and free radicals, thereby reducing inflammation and preventing cellular damage [79]. Both oxidative and inflammatory processes are thought to be involved in several different pathogenesis mechanisms [77,80,81,82,83,84]. Antioxidants exert their protective effects through a combination of direct scavenging of free radicals, modulation of enzyme activity, induction of antioxidant defenses, and modulation of signaling pathways. These mechanisms collectively contribute to the ability of antioxidants to mitigate oxidative stress and prevent cellular damage associated with aging, inflammation, and various diseases [85,86].

It is crucial to explore and develop new medicines to address these health challenges. The discovery of novel molecules exhibiting antioxidant activity is essential for advancing innovative medical solutions that contribute to global well-being.

Recently, Martins et al. [49] assessed the antioxidant activity of hydroethanolic extracts from eight macrofungi. *P. tinctorius* stood out as one of the fungi with the highest values of antioxidant activity, evaluated by three different methods: 2,2-di(4-tertoctylphenyl)-1-picrylhydrazyl (DPPH), 2,20-azino-bis(3-ethylbenzothiazoline-6-sulfonic acid) (ABTS), and ferric reducing antioxidant power (FRAP), with 1291.00, 519.10 and 128.30 µM Trolox (6-hydroxy-2,5,7,8-tetramethlychroman-2-carboxylic acid)/g, respectively (Table 3). 

Similarly, Pringle et al. [87] showed that *P. tinctorius* ethanolic and aqueous extracts had the highest antioxidant activity among four tested fungi. It is interesting to note that the *P. tinctorius* ethanolic extract, at 200 µg/mL, had a similar antioxidant activity, assessed by DPPH, as catechin, a reference compound with high antioxidant capacity.

The high antioxidant activity of *P. arhizus* in comparison with other macrofungi was also demonstrated in the study of Campi et al. [88]. The antioxidant concentration determined by DPPH was 36.66 mg/g of ascorbic acid equivalent (AAE), the second highest among nine tested fungi. 

The collective findings from these recent studies show high antioxidant activity across diverse assessments, including DPPH, ABTS, and FRAP methods, positioning *Pisolithus* as a promising source of antioxidant compounds.

Reis et al. [89] evaluated the antioxidant activity of methanolic extracts of *P. arhizus* using three different methods. Interestingly, all methods revealed that the antioxidant activity of *P. arhizus* varied among its different fungal components. In basidiocarps, the antioxidant activity assessed by DPPH radical scavenging activity, reducing power, and inhibition of β-carotene bleaching was EC_50_ 0.56, 0.37, and 0.24 mg/mL, respectively. In mycelium, it was EC_50_ > 20.00, 7.29, and 2.49 mg/mL, respectively, showing that basidiocarps have higher antioxidant activity than mycelium.

## 3. Conclusions

In the exploration of *Pisolithus*, this comprehensive review enlightens the bioactive potential harbored within the genus, offering insights into its antimicrobial, anticancer, and antioxidant properties. Although bioactive compounds isolated from *Pisolithus* have been understudied, the bioactive potential of the genus is promising and deserves to be compiled to stimulate further investigations.

Three species of *Pisolithus* that exhibited bioactive potential have been reported, namely *P. arhizus*/*P. tinctorius*, *P. albus* and *P. microcarpus*. The diverse bioactive potential of *Pisolithus* is underscored by the discovery of compounds with markedly distinct chemical structures, extracted from different fungal structures—be it the basidiocarp or the mycelium, and the potential hidden in other structures such as the spores. This not only highlights the rich reservoir of bioactive compounds within *Pisolithus* but also accentuates the importance of exploring the multifaceted contributions of different fungal components in unlocking innovative applications across diverse therapeutic landscapes.

Compounds derived from *Pisolithus* exhibited antimicrobial activity against a spectrum of microorganisms, showcasing efficacy against both Gram-negative and Gram-positive bacteria, mycobacteria, dermopathogenic fungi, phytopathogenic fungi, and phytopathogenic oomycetes. Particularly noteworthy is the effectiveness of *Pisolithus* against antibiotic-resistant bacteria, positioning it as a valuable contender in the battle against antibiotic resistance. The bioactive compounds from *Pisolithus* demonstrated antimicrobial performance comparable to established antibacterial and antifungal medications, accentuating the therapeutic potential of these natural products. Their ability to match the efficacy of current pharmaceuticals is a testament to the capacity of *Pisolithus*-derived compounds.

In the realm of anticancer activity, the triterpene pisosterol emerges as a standout compound from *P. tinctorius*. With documented cytotoxicity against various cancer cell lines, pisosterol holds promise as a novel candidate for anticancer therapies. Despite significant strides in understanding its effects in vitro and in vivo, further investigations are warranted to unravel the mechanisms underlying its anticancer properties.

Furthermore, *Pisolithus* has been unveiled as a potential source of antioxidants, with studies on extracts and fractions showcasing promising results. Notably, basidiocarps exhibited superior antioxidant activity compared to mycelium, offering avenues for targeted extraction and isolation strategies.

While in vitro studies presented compelling evidence of the *Pisolithus* bioactive prowess, it is imperative to acknowledge the necessity for in vivo validation. Limited information on isolated compounds and a selective focus on certain activities underscore the need for expansive studies encompassing a broader spectrum of compounds and activities. Mechanistic insights into the mode of action of *Pisolithus*-derived compounds remain a compelling area for future research.

Most of the compounds isolated from *Pisolithus* belong to chemical groups well known for their bioactive potential. Pisolithin A and B are phenolic compounds. Phenolics exhibit a range of bioactivities, including antimicrobial, anticancer, and antioxidant effects [93]. Pisolactone and 7,22-dien-3-ol, 24-methyl lanostane are two triterpenoids, and this class of organic compounds has been described as potential antimicrobial and anticancer drugs [94,95]. Pisosterol is a triterpene. Triterpenes were found to induce antimicrobial, antiviral, anticancer, and antioxidant properties, among others [96,97,98]. The chemical nature of the isolated compounds thus reflects what is already known regarding their therapeutic potential and elevates *Pisolithus* to another level on the scale of pharmacological interest. While producing biomass for compound isolation at an industrial scale may pose challenges, natural models can serve as valuable tools for designing novel agents with potent bioactivities. Therefore, compounds from *Pisolithus* may continue to represent promising leads for the development of new drugs in the future [95].

As our understanding of the multifaceted nature of *Pisolithus* continues to evolve, the wealth of bioactive potential residing in *Pisolithus* signifies a promising frontier in the development of innovative antimicrobial, anticancer, and antioxidant applications for addressing contemporary health challenges. 

## Figures and Tables

**Table 3:** Extracts of *Pisolithus* species with antioxidant activity.

Extract	Species	Fungal Structure	Assay	Reference
Hydroethanolic	*P. tinctorius*	Basidiocarp	DPPH radical scavenging activity ABTS radical scavenging activity FRAP	[49]
Ethanolic Aqueous	*P. tinctorius*	Basidiocarp	DPPH radical scavenging activity FRAP	[87]
Ethanolic	*P. arhizus*	Basidiocarp	DPPH radical absorbance	[88]
Methanolic	*P. arhizus*	Basidiocarp Mycelium	DPPH radical scavenging activity Reducing power Inhibition of β-carotene bleaching	[89]
Hydroethanolic Methanolic	*P. tinctorius*	Basidiocarp	DPPH radical absorbance	[90]
Butanolic Ethyl acetate	*P. microcarpus*	Mycelium	DPPH radical scavenging activity	[47]
Ethanolic	*P. arhizus*	Basidiocarp	Total antioxidant status (TAS) Total oxidant status (TOS) Oxidative stress index (OSI)t	[91]
Ethanolic	*P. albus*	Basidiocarp	DPPH radical scavenging activity Reducing power of iron iron-chelating power	[48]
Methanolic Ethanolic	*P. arhizus*	Basidiocarp	DPPH radical scavenging activity Metal chelating power	[92]

DPPH—2,2-di(4-tertoctylphenyl)-1-picrylhydrazyl; ABTS—2,20-azino-bis(3-ethylbenzothiazoline-6-sulfonic acid); FRAP—ferric reducing antioxidant power.

## Data Availability

Not applicable.
